# Physical activity and successful aging among middle-aged and older adults: a systematic review and meta-analysis of cohort studies

**DOI:** 10.18632/aging.103057

**Published:** 2020-04-29

**Authors:** Yi-Hsuan Lin, Yi-Chun Chen, Yen-Chiang Tseng, Shih-tzu Tsai, Yen-Han Tseng

**Affiliations:** 1 Department of Family Medicine, Cheng Hsin General Hospital, Taipei, Taiwan; 2 Department of Family Medicine, School of Medicine, National Yang-Ming University, Taipei, Taiwan; 3 Department of Public Health, College of Public Health, National Taiwan University, Taipei, Taiwan; 4 Division of Thoracic Surgery, Department of Surgery, Kaohsiung Veterans General Hospital, Kaohsiung, Taiwan; 5 Institute of Clinical Medicine, National Yang-Ming University, Taipei, Taiwan; 6 School of Medicine, National Yang-Ming University, Taipei, Taiwan; 7 Division of Pulmonary Medicine, Department of Internal Medicine, Shuang Ho Hospital, Taipei Medical University, Taipei, Taiwan

**Keywords:** successful aging, healthy aging, physical activity, exercise, older people

## Abstract

Background: We aimed to investigate the association between physical activity and successful aging among middle-aged and older adults and study how this association changes with age and time.

Results: The mean score of Newcastle-Ottawa Scale assessment was 8.0±0.8. Physically active middle-aged and older adults were more likely to age successfully than sedentary adults (OR=1.64, 95%CI: 1.40–1.94). The effect of physical activity was stronger in the younger group (OR=1.71, 95%CI: 1.41–2.08) than on the older group (OR=1.54, 95%CI: 1.13–2.08). However, the protective effect of physical activity reduced annually by approximately 3%.

Conclusions: Physical activity promotes successful aging among middle-aged and older adults especially in the younger population. Being physically active at middle and old age is beneficial to successful aging.

Methods: We searched for the relevant studies in three online databases: Pubmed, Web of Science, and Embase. Fifteen community-based cohort studies were included. The Newcastle-Ottawa Scale assessment Form was used for quality assessment. Overall, 189,192 participants aged 43.9-79.0 years were analyzed. The odds ratio for successful aging of the most physically active group compared with sedentary group was analyzed. Subgroup analysis was conducted by age group. Univariate Meta-regression was performed according to follow-up years.

## INTRODUCTION

Aging is a global problem. According to the World Health Organization’s report about global health and aging, the number of people aged ≥ 65 will increase to approximately 1.6 billion in 2050 and comprise 16% of the world’s population [1]. Aging is associated with health-related problems and substantial medical cost. Disease patterns among the elderly also shift to chronic non-communicable diseases such as cardiovascular disease, hypertension, diabetes, cancer, and dementia [1]. In the US, England, and Europe, approximately 43%–59% of adults aged 50–74 years experienced more than one mobility impairment [2]. Aging with disability demands long-term care [3]. The estimated duration of long-term care for Americans is approximately 2 years, and one-seventh of the US population need long-term care for more than 5 years [4]. There is a global trend of increase in the demand for long-term care. This means that people will experience a period of disability and dependence on others in their daily living before death.

Studies about successful aging have emerged in the past 20 years. Older adults who aged successfully maintain their function and experience morbidity and disability for a shorter period [5]. Successful aging means preserving life quality and reducing the health burden caused by aging [6]. Successful aging was proved to decrease the risk of long-term care [7]. Successful aging is a multidimensional concept and overlaps with “healthy aging,” “aging well,” and “positive aging” [8, 9]. In 1997, Rowe and Kahn proposed the biomedical theories of successful aging [10]. The biomedical theory included three components: the absence of disease and related risk factors, maintenance of physical and cognitive function, and active engagement with life. While the biomedical theory emphasized physical and cognitive functions, the psychosocial model highlighted life satisfaction and well-being, social participation and activity, personal growth, and psychological resources [11]. Additional views of successful aging included accomplishments, enjoyment of diet, financial security, neighborhood, and physical appearance [11]. Some review articles tried to organize the definition of successful aging into three domains: physiological, psychological, and social domains [12]. At present, successful aging is a complete and multi-aspect concept.

Many factors affect successful aging, including physiological, psychological, social, and lifestyle factors [13, 14]. Physical activity is an important lifestyle factor that can delay the onset of chronic diseases [15, 16], increase longevity and survival [17, 18], and improve cognitive and physical functions in the older people [19, 20]. However, previous studies on physical activity and successful aging reported inconsistent results. Some studies observed a strong correlation between physical activity and successful aging [21–23], while others showed a weak association [24–26].

Although previous a meta-analysis [27] integrated existing evidences to clarify the association between physical activity and successful aging, to our knowledge, no article has focused on middle-aged and older adults. In addition, studies discussing the effects of time on the association between physical activity and successful aging are also limited. Thus, the primary aim of this meta-analysis was to investigate the association between physical activity and successful aging for the middle-aged and older adults. The secondary aim was to demonstrate the effects of age and time. We hoped to shed light on how to promote successful aging in the global aging society.

## RESULTS

### Description of studies and quality assessment

**Figure 1:**
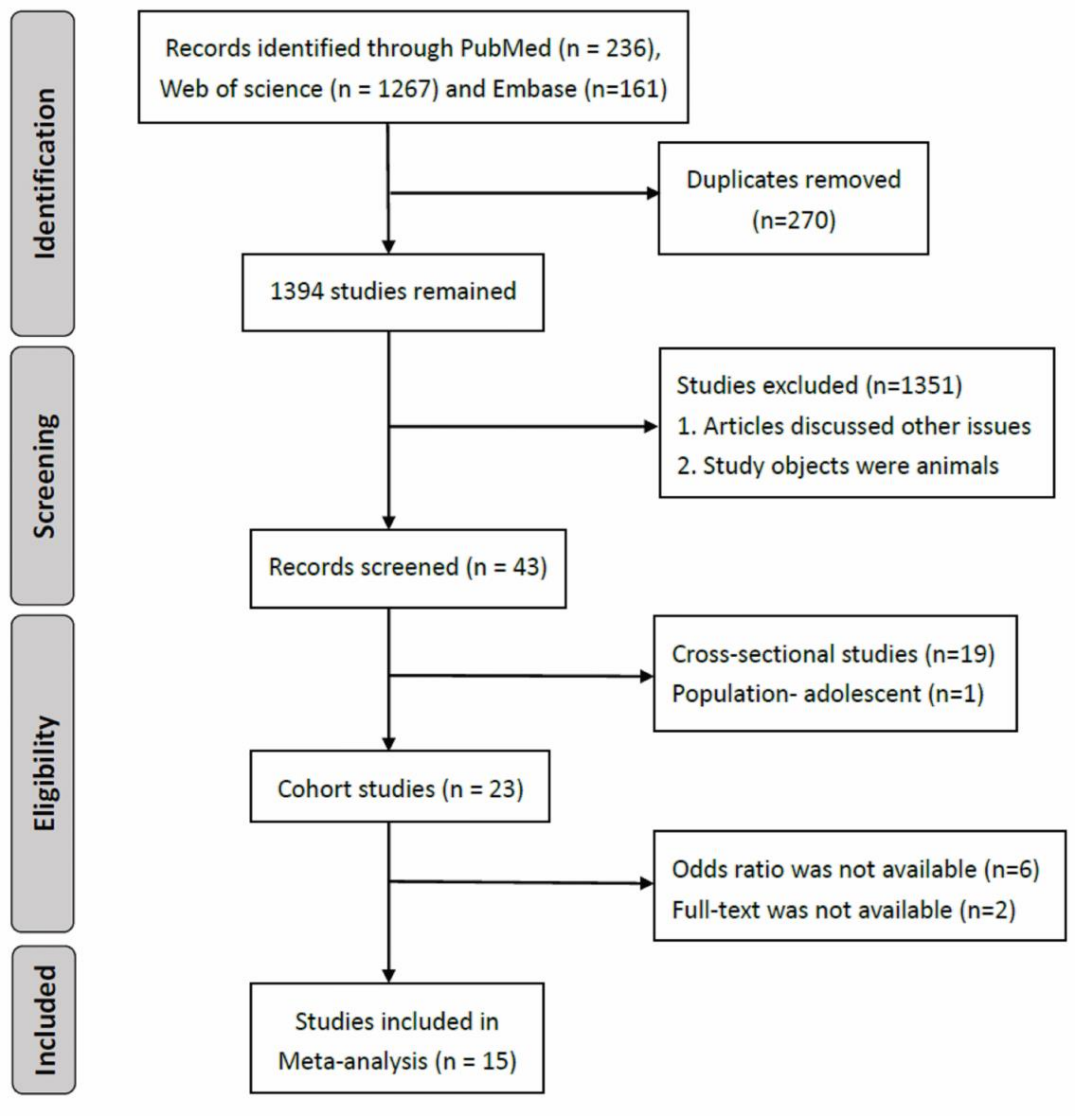
**Flow chart of search strategy.**

We obtained 1,664 articles through the three research databases. Among the 1,394 articles without duplicate, only 43 discussed the association between physical activity and successful aging. To identify the causal inference of physical activity and successful aging, we excluded 19 cross-sectional studies. Finally, 15 cohort studies were included for analysis (Figure 1).

Among the included articles, 10 were published in the recent 10 years (Table 1). The study population included older populations from Australia, United States, Britain, Europe, Nigeria, Cuba, Dominican Republic, Peru, Mexico, and Puerto Rico. The total sample size at baseline was 189,192. The mean age of participants ranged from 43.9-79.0 years. Participants of the seven studies were a mixture of middle-aged and older people. The follow-up duration in 10 studies was > 10 years.

**Table 1:** Characteristics of included studies.

**Study (Year)**	**Population**	**Age range (Baseline)**	**Mean age (Baseline)**	**Follow-up years**	**Follow-up rate (%)**	**Sample Size (Baseline)**	**Men (%)**	**Percentage of successful aging**	**Quality Score**
Gopinath (2018)	Australia	≥ 49	65.2	10	62.4	3,654	43.4	15.7	9
Daskalopoulou (2018)	Cuba, Dominican Republic, Peru, Mexico and Puerto Rico	≥ 65	74.2	4	66.4	10,900	33.8	15.1	8
LaCroix (2016)	United States	50-79	68.9	20	77.1	88,404	0	31	7
Almeida (2014)	Australia	65-83	72.1	11	43.6	12,201	100	11.7	6
Bell (2014)	American men of Japanese ancestry	72-82	75.7	21	77.4	1,292	100	34	6
Gureje (2014)	Nigeria	≥ 65	79	5	44.5	2,149	61.1	7.5	8
Hodge (2014)	Australia	≥ 57	64.1	13	74	25,607	38.6	18.6	8
Hamer (2014)	British	≥ 50	63.7	8	33.7	11,391	42.5	19.3	7
Sabia (2012)	British	35-55	51.3	18	77.3	6,599	70.5	18.7	8
Sun (2010)	United States	40-65	60	14	>95	13,535	0	10.8	8
Kaplan (2008)	Canada	65-85	72.6	10	88.8	2,740	40.5	7.8	8
Britton (2008)	British	35-55	43.9	17	78.6	7,410	71.1	13.6	7
Haveman-Nies (2003)	Europe	70-75	72.5	10	58.7	2,200	49.6	28.8	7
Ford (2000)	United States	≥ 70	77.5	2	80.9	602	29.7	20.1	6
Strawbridge (1996)	United States	≥ 65	71.9	6	70.1	508	41	35	7

The criteria of physically active group differed among studies (Supplementary Table 1). Some studies used standard rating scale of physical activity such as international physical activity questionnaire [28], physical activity index [25], or Voorrips score [5]. Other studies used questionnaires to classify the level of physical activity. The included studies had varied definitions of successful aging. Most studies followed the biomedical model [10] that defines successful aging as the absence of chronic diseases and preservation of physical and cognitive functions. In eight studies [21, 22, 28–33], the rate of successful aging range from 10% to 20% (Table 1). Two cohort studies reported relatively low rate of successful aging. Kaplan et al. [34] reported 7.8% and Gureje et al. [35] reported 7.5% of successful aging.

Some studies recruited a special population. For example, Bell et al. [25] reported life factors associated with successful aging in American men of Japanese ancestry. LaCroix et al. [36] reported the predictors of successful aging for postmenopausal female veterans. Sun et al. [21] investigate the association of physical activity at midlife with successful survival for female registered nurses. Almeida et al. [30] investigated successful aging in older men. In other included studies, the participants were general population from communities. For the quality assessment, the quality score of the included studies, assessed by the Newcastle-Ottawa Quality Assessment Form, ranged from 7 to 9 (mean score: 8.0±0.8, Table 2).

**Table 2:** The quality assessment of included studies by the Newcastle-Ottawa Quality Assessment Form for cohort studies.

**Study (Years)**	**Selection**			**Comparability**			**Outcome**		**Total quality**
	**Representativeness of the exposed cohort**	**Selection of the non-exposed cohort**	**Ascertainment of exposures**	**Demonstration that outcome of interest was not present at start of study**	**Comparability of cohorts on the basis of the design or analysis controlled for confounders**	**Assessment of outcome**	**Was follow up long enough for outcomes to occur?**	**Adequacy of follow up of cohort**	
Acceptable	From community of general population	From the same community as exposed cohort	From structured interview	Yes	Yes, at least age and sex	Contained objective indicators	At least 4 years	Follow up rate more than 80%, or subjects lost to follow up unlikely to introduce bias	
Gopinath (2018)	1	1	1	1	2	1	1	1	9
Daskalopoulou (2018)	1	1	0	1	2	1	1	1	8
LaCroix (2016)	0	1	1	1	2	1	1	1	8
Almeida (2014)	0	1	1	1	2	1	1	0	7
Bell (2014)	0	1	1	1	1	1	1	1	7
Gureje (2014)	1	1	1	1	2	1	1	0	8
Hodge (2014)	1	1	0	1	2	1	1	1	8
Hamer (2013)	1	1	1	1	2	1	1	0	8
Sabia (2012)	1	1	1	1	2	1	1	1	9
Sun (2010)	0	1	1	1	2	1	1	1	8
Kaplan (2008)	1	1	1	1	2	1	1	1	9
Britton (2008)	1	1	1	1	2	1	1	1	9
Haveman-Nies (2003)	1	1	1	1	2	1	1	0	8
Ford (2000)	1	1	0	1	2	1	0	1	7
Strawbridge (1996)	1	1	0	1	1	1	1	1	7

### Overall effect size

**Figure 2:**
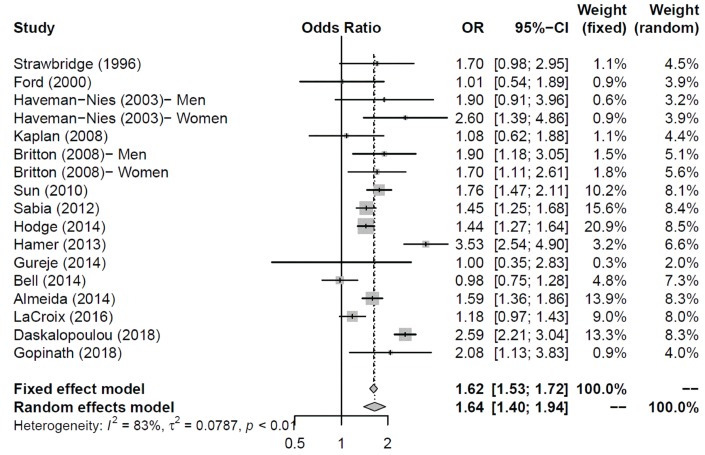
**The overall effect (odds ratio) of physical activity to successful aging.**

The overall odds ratio (OR) of physical activity to successful aging was 1.64 [95% confidence interval (CI) = 1.40–1.94] in the random-effects model (Figure 2). I^2^ (83%) revealed a high heterogeneity among the included studies. We further performed Egger’s test, and its *p-*value was 0.87. Thus, the publication bias did not exist at the 5% significant level. Although publication bias was not obvious in Egger’s test, the trim-and-fill method was still performed. However, no study was filled by the trim-and-fill method.

### Subgroup analysis

**Figure 3:**
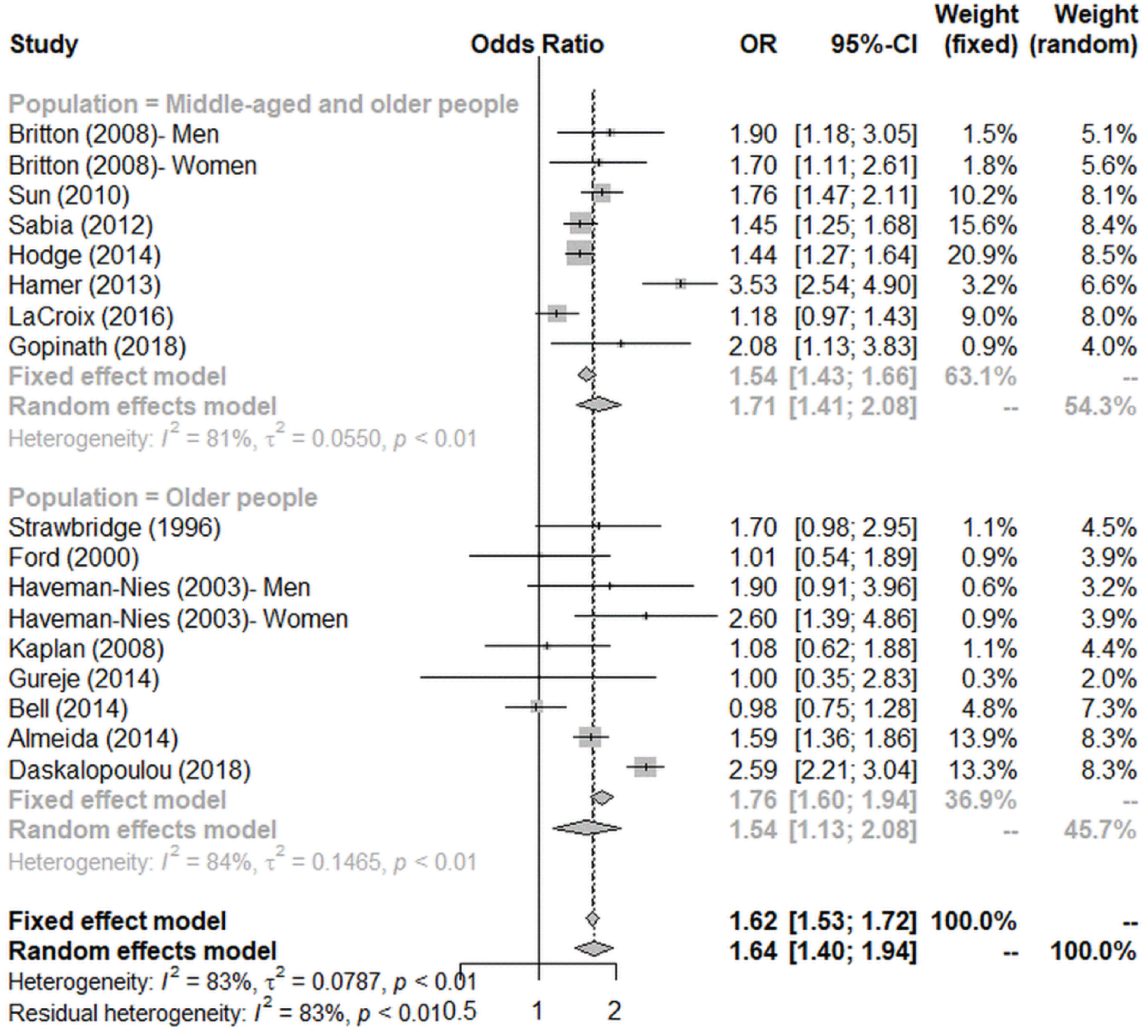
**Subgroup analysis by age on the association between physical activity and successful aging.**

Subgroup analysis (Figure 3) was conducted according to the age group of participants. When all the participants aged >65 years, the effect size became smaller (OR = 1.54, 95% CI = 1.13–2.08). On the contrary, we observed a larger effect in studies in which the recruited participants were a mixture of middle-aged and older adults (OR = 1.71, 95% CI = 1.41–2.08).

### Meta-regression analysis

**Figure 4:**
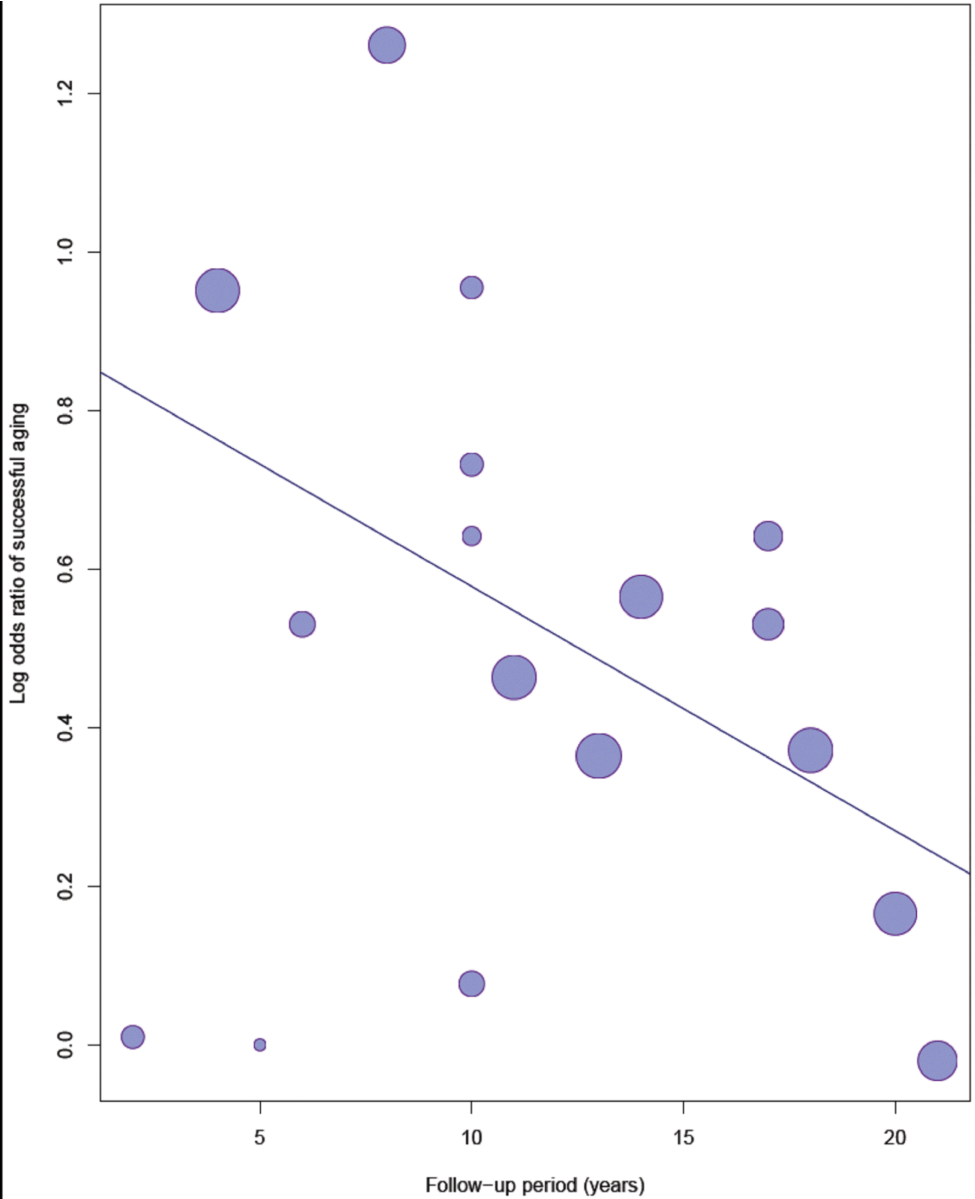
**Meta-regression analysis of the log odds ratio of physical activity to successful ageing and follow-up years.**

Figure 4 shows the bubble plot with fitted meta-regression line of the log OR of successful aging and follow-up years. In the univariate regression model, the regression line showed a significantly decreased trend of successful aging over time (OR= 0.97, 95% CI = 0.94–0.99, *p* = 0.045). The effect of physical activity on successful aging reduced annually by approximately 3%.

## DISCUSSION

This meta-analysis showed a protective effect of physical activity to successful aging among the middle-aged and older adults. The protective effect of physical activity to successful aging was larger on the younger group than the older group. Being physically active in earlier life is beneficial to successful aging in later life. However, the effect of physical activity on successful aging decreased as time elapsed.

Physical activity prevents the development of many chronic diseases, including metabolic syndrome, type 2 diabetes, coronary artery disease, hypertension, stroke, dyslipidemia, cognitive impairment, depression, osteoarthritis, osteoporosis, colon cancer, breast cancer, non-alcoholic fatty liver disease and sarcopenia [37]. Physical activity also increases longevity and survival [5, 38]. For middle-aged and older people, a dose-response relationship was found between physical activity and decrease in mortality [38]. Compared with sedentary older people, physically active older adults were more likely to remain living independently [17]. Physical activity in old age preserves the cognitive and physical functions [17, 39]. These previous findings supported the main finding of the present meta-analysis.

Physical activity is a protective factor of successful aging in the middle-aged and older adults. Although some included studies showed a weak association between physical activity and successful aging [24–26, 34–36], most studies reported a consistent positive relationship. Some studies with insignificant results [25, 36] included a specific population. Thus, the representativeness of the exposed cohort might be limited. Besides, the use of a very simple questionnaire to classify physical activity might lead to misclassification bias of exposure and influence the results [26]. The ratio of successful aging was relatively low in two studies. The rigorous definition of successful aging contributed to the low percentage of successful aging and reduced the effect of physical activity. Gureje et al. [35] defined successful aging as the absence of chronic diseases, including hypertension. Nevertheless, hypertension is very common in older people. In a survey for non-institutionalized population in England, US, and Canada, the prevalence of hypertension for older people aged 60-80 years was 63.7%, 63.6%, and 53.2%, respectively [40]. The strict definition of successful aging might result in the null effects of physical activity on successful aging.

Our main results were consistent with the past meta-analysis about physical activity and successful aging. Daskalopoulou et al. reported that physical activity had an effect size of 1.39 (95% CI = 1.23–1.57) on healthy aging [27]. In our study, the effect size of physical activity on successful aging was stronger because the included studies were not the same. Our results showed that the protective effects of physical activity to successful aging decreased over time. Time plays an important role in the aging process [41]. Thus, how to reduce the influence of time and preserve the benefits of physical activity would be issues we need to focus on. Further research is warranted if the effects of time could be attenuated by increasing the intensity of physical activity or combining other protective factors.

High heterogeneity was observed among the included studies. The diversity was caused by the different definitions of physical activity and successful aging. Unlike some diseases with diagnostic guideline, there are no standard criteria for defining successful aging. Although previous review articles [12, 13, 42] tried to organize the components of successful aging, researchers engaging in successful aging still used different domains and different weightings of the domains. The diversity resulted in the heterogeneity of successful aging in our analysis.

The criteria for grouping of physical activity differed among studies. Because most studies did not provide the quantitative data of physical activity, further analysis of the dose-response relationship between physical activity and successful aging is challenging. In our study, we used the effect size of the most vigorous activity group compared with the most sedentary group in each study to perform the meta-analysis. We could not conclude a quantitative suggestion of physical activity to successful aging for middle-aged and older adults. For the recommendations of physical activity for adults aged >65, WHO suggested that older adults should perform at least 150 min of moderate-intensity or 75 min of vigorous-intensity physical activity per week to improve cardiorespiratory function and muscle fitness [43].

The present study has some limitations. First, we included cohort studies, but not randomized control trails (RCTs). RCTs are undoubtedly the golden standard of study designs to clarify causal-inference. However, for physical activity and successful aging, it is difficult to blind the participants to the exposure (physical activity). Furthermore, the contamination effects in RCTs unavoidably occur with time. Current RCTs discussing the effects of physical activity on successful aging have short follow-up period and the small sample size [44, 45]. Well-designed observational studies were quite suitable for the discussions of such issue. Among the non-experimental study designs, cohort studies established a clear temporality to confirm the causal inference between exposure and outcome. Therefore, we included only cohort studies and excluded cross-sectional studies. Most of our included studies enrolled a representative population from the community. Thus, the generalizability of our results was feasible. Second, the dose-response relationship between physical activity and successful aging could not be established. Because the included studies lacked quantitative data of physical activity, we can only conclude that physical activity promotes successful aging in middle-aged and older adults. The dose-response relationship can be analyzed only if future studies used quantitative grouping of physical activity such as metabolic equivalents. Third, publication bias was possible. Studies with the null results might not be published. Studies not enrolled in Pubmed, Web of Science, and Embase as well as studies without using the keywords we used in our search process might be omitted. However, we conducted the Egger’s test, and the potential publication bias was not significant.

Despite these limitations, our study was the first meta-analysis to analyze the association between physical activity and successful aging especially for the middle-aged and older adults. In addition, we found that the protective effect of physical activity to successful aging attenuated over time. Our study contributed to the discussions of public health policy in the global aging society. The main focus of health promotion policy in the aging society is to encourage middle-aged and older adults to be physically active. Future research should aim at the quantitative suggestions of physical activity to reduce the effects of time.

## CONCLUSIONS

This meta-analysis found a positive effect of physical activity to successful aging in middle-aged and older adults. However, the effect attenuated over time. Further research is warranted to establish the dose-response relationship between physical activity and successful aging as well as to reduce the effects of time.

## MATERIALS AND METHODS

### Search strategy

Literature review was conducted in the online databases of PubMed, Web of Science, and Embase from May 6, 2019, to July 5, 2019. We used the keywords “successful aging,” “healthy aging,” and “aging well” in the title and “physical activity” in all searching fields. We further limited the article type to original article and language to English. Cohort studies discussing the association of physical activity and successful aging were eligible for our analysis. Articles were excluded if (1) studies discussed other issues, (2) research objects were not humans, (3) the study design was cross-sectional, (4) the study population was not middle-aged or old people, (5) OR was not available, (6) full-text article was not available, and (7) studies other than original research such as review article, meta-analysis, letters, or case series. If two studies used the same data source and adjusted finely in methods, only one article was adopted.

### Data extraction

The following information was extracted from the included studies: study population, baseline age, follow-up years, follow-up rate, sample size, proportion of men, rate of successful aging, definition of physical activity and successful aging, adjusted covariates, and treatment effects (OR). For studies that classified physical activity to more than two groups, the effect size of the most vigorous physically active group compared with the most sedentary group was considered for analysis. For studies that provided the OR of men and women separately, we considered the effects of both sexes in the overall analysis.

### Quality assessment

We used the Newcastle-Ottawa Quality Assessment Form for Cohort Studies [46] to evaluate the quality of the included studies. The scale included three sections and eight items as follows: (1) representativeness of the exposed cohort, (2) selection of the non-exposed cohort, (3) ascertainment of exposure, (4) demonstration that the outcome of interest was not present at the start of the study, (5) comparability of cohorts on the basis of the design or analysis controlled for confounders, (6) assessment of outcome, (7) follow-up was long enough for outcomes to occur, (8) adequacy of follow-up of cohorts. The total score was 9 stars. Quality assessment was performed by two investigators. In case of differing opinions, a consensus was reached by discussion.

### Data analysis

Statistical analysis was performed using R version 3.5.1 (R Foundation for Statistical Computing, Vienna, Austria). The overall effect was shown by the forest plot. As the included studies had different study population and design, we used the random-effects model to incorporate the heterogeneity [47]. We perform Egger’s test [48] to examine possible publication bias. Subgroup analysis was conducted to evaluate the effect size of different age groups. Meta-regression was then performed to show the effect of follow-up years. A *p*-value of <0.05 (two-tailed) was considered statistically significant.

## Supplementary Material

Supplementary Table 1
