# Supplementary material for: Physical activity and successful aging among middle-aged and older adults: a systematic review and meta-analysis of cohort studies
Source: Aging (Albany NY). 2020 Apr 29;12(9):7704–16. doi: 10.18632/aging.103057 (PMC7244057; doi:10.18632/aging.103057)
Supplement: Supplementary Table 1 [file aging-12-103057-s001..docx]

**Supplementary Table 1. Definition of physical activity, successful aging and adjusted covariates of included studies.**

| **Study (Year)/ Study Project** | **Outcome/ Assessment Tool** | **Classification of Physical Activity** | **Adjusted Covariates** |
| --- | --- | --- | --- |
| Gopinath (2018)/ Blue Mountains Eye Study | Absence of stroke, coronary artery disease, angina, acute myocardial infarction, cancer, or diabetes; optimal cognitive, physical, mental, respiratory and vascular function; and the lack of disability; and being functionally independent. | International Physical Activity Questionnaire- metabolic equivalents (METs) over 1 week. Physical active: METs≥5000 Physical inactive: METs <1000 | Age, sex, marital status, smoking, BMI and living status |
| Daskalopoulou (2018)/ 10/66 Dementia Research Group | Healthy ageing index (26 health related question-indicators, including information on daily disabilities and difficulties, pain and sleep problems, cognition abilities) quintiles of the baseline healthy aging score,  Normal agers: three lowest fifths (i.e., 0–67.92 scores) Healthy agers: two highest fifths (i.e., 67.93–100 scores) | Questionnaire Physical active: "very physically active" and "fairly" Physical inactive: "not very" and "Never" | Age, sex, smoking, alcohol, education, consumption of fruits and vegetables |
| LaCroix (2016)/ Women's Health Initiative | Lived to age 80 years; no major disease (coronary disease, stroke, cancer, hip fracture, and type-2 diabetes); no mobility disability | Self-reported duration and frequency of exercise-metabolic equivalent hours per week (MET-hours/week) Physical active: >10.5 MET-hours/week Physical inactive: ≤10.5 MET-hours/week | Age, race, marital status, smoking, alcohol, education, BMI, family income, hormone use and depression |
| Almeida (2014)/ Health In Men Study | No depressive symptoms, preserved cognition and  daily physical function | Questionnaire Physically active: 150 min or more per week in vigorous activity Physical inactive: less than 150 min | Age, marital status, smoking, alcohol, education, BMI, hypertension, diabetes, coronary heart disease and cerebrovascular disease at baseline. |
| Bell (2014)/ Honolulu Heart Program | Survived to age 85 years and free from the chronic diseases (coronary heart disease, stroke, cancer, chronic obstructive pulmonary disease, Parkinson's disease and treated diabetes mellitus); no physical impairment; no cognitive impairment. | Physical activity index (PAI)- Physically active: PAI >30.4 Physical inactive: PAI ≤30.4 | Age |
| Gureje (2014)/ Ibadan Study of Aging | Absence of chronic health condition (Hypertension, arthritis, diabetes, heart disease, asthma, depression, dementia); complete functional independence; self-reported satisfaction with life | International Physical Activity Questionnaire-  Physical active: vigorous levels of physical activity Physical inactive: low levels of physical activity | Age, sex, smoking, alcohol, education, residence, economic status, self-reported health, contact with family, contact with friends, participation in household activities, participation in community activities, functional disability at baseline |
| Hodge (2014)/ Melbourne Collaborative Cohort Study | Surviving to age 70 years or older; absence of major chronic diseases; no major limitations of physical function and maintaining good mental health | Questionnaire Physical active: moderate-high physical activity Physical inactive: none-low physical activity | Age, sex, marital status, smoking, alcohol, education, BMI, follow-up period, diet, waist to hip ratio, asthma, hypertension, arthritis, kidney stones, gallstones |
| Hamer (2014)/  English Longitudinal Study of Ageing | Being free from major chronic disease; preserve cognitive function and physical functions; good mental health. | Questionnaire Vigorous physical active: vigorous activity at least once a week Physical inactive: no moderate or vigorous activity in a week | Age, sex, marital status, smoking, alcohol, economic status. |
| Sabia (2012)/ Whitehall II cohort study | No disease of cancer, coronary artery disease, stroke or diabetes; good cognitive, physical, respiratory and cardiovascular function; absence of disability; good mental health. | Questionnaire Physical active: ≥2.5 hours/week moderate physical activity or ≥1 hours/week vigorous physical activity Physical inactive: no physical activity | Age, sex, marital status and education |
| Sun (2010)/ Nurses' Health Study | No history of cancer, diabetes, myocardial infarction, coronary artery bypass graft surgery, congestive heart failure, stroke, kidney failure, chronic obstructive pulmonary disease, Parkinson's disease, multiple sclerosis, or amyotrophic lateral sclerosis; No impairment in cognitive function; No physical disabilities; No mental health limitations | Questionnaire The average time per week in the past year participants spent on leisure-time physical activities- metabolic-equivalent tasks (METs) Physical Activity Quintile  Physical active: MET ≥22.9 (h/wk) Physical inactive: MET 0.2-2.3 (h/wk) | Age, marital status, smoking, alcohol, education, BMI, hormone use, family history of heart disease, diabetes, or cancer, dietary polyunsaturated to saturated fat ratio, intakes of trans fat, and cereal fiber; and intakes of fruits and vegetables and red meat. |
| Kaplan (2008)/ Canadian National Population Health Survey | Health Utilities Index Mark 3 (HUI3): vision, hearing, speech, ambulation, dexterity, emotion, cognition, and pain/discomfort Successful aging group: HUI3 score ≥0.89 at each cycle Non-successful aging group: HUI score <0.89 and not institutionalized surviver | Questionnaire Physically active: at least 3 times per week for at least 15 minutes Physically inactive: less than 3 times per week for at least 15 minutes | Age, sex, marital status, smoking, alcohol, education, family income, psychosocial factors, normal weight, health status |
| Britton (2008)/ Whitehall II cohort study | Free from major disease (coronary artery disease, stroke, cancer, diabetes mellitus, depression, or metabolic syndrome); good physical and mental function | Questionnaire Vigorous physical active: >1 hours/week physical activity Physical inactive: no physical activity | Age, socio-economic position and number of phases attended |
| Haveman-Nies (2003)/ European multi-centre SENECA study | Remain physical independent | Physical Activity Questionnaire for the Elderly (Voorrips score)-Sex-specific tertiles Physical active: the intermediate- and high activity tertiles Physical inactive: the low-activity tertile | Age, sex, smoking, education, BMI, dietary quality |
| Ford (2000) | Physical independence: activities of daily living and instrumental activities of daily living | Questionnaire: Do you exercise regularly? Physical active: yes Physical inactive: no | Age, sex, race, marital status, smoking, alcohol, education, adequate income, physical function, medical condition, cognitive status, depression, attitudes |
| Strawbridge (1996)/  Alameda County Study | Normal basic physical activities, no more than a little difficulty doing physical performance activities | Questionnaire: Do you often walk for exercise? Physical active: yes Physical inactive: no | Age, sex and baseline successful aging |

Abbreviations: BMI: Body Mass Index; MET: Metabolic Equivalent; PAI: Physical Activity Index.
